# Parametric evaluation of procedural fidelity level with combined errors during differential reinforcement of other behavior

**DOI:** 10.1002/jaba.70077

**Published:** 2026-08-17

**Authors:** Paige O'Neill, Catalina N. Rey, Thomas Eilers, Andrew R. Craig

**Affiliations:** ^1^ Integrated Center for Autism Spectrum Disorders University of Nebraska Medical Center's Munroe‐Meyer Institute Omaha NE USA; ^2^ School of Psychological and Behavioral Sciences Southern Illinois University Carbondale Carbondale IL USA; ^3^ Behavior Analysis Studies, Pediatrics, and Neuroscience and Psychology Departments, Golisano Center for Special Needs SUNY Upstate Medical University Syracuse NY USA

**Keywords:** differential reinforcement of other behavior, human operant, parametric evaluation, procedural fidelity, translational research

## Abstract

Differential reinforcement of other behavior (DRO) is an intervention used to address challenging behavior; however, its effectiveness may be diminished by procedural fidelity errors. Previous research has shown that DRO effects may be robust despite marginal errors of commission and omission, but the effects of combined errors (i.e., both commission and omission errors) on the efficacy of DRO have not been investigated. The purpose of this study was to parametrically evaluate procedural fidelity level (i.e., 100, 80, 60, 40, and 20% fidelity) in DRO when implemented with combined errors in a human‐operant arrangement using a reversal design. Results indicated that the DRO was efficacious for most participants when implemented with 100, 80, or 60% fidelity and inefficacious for most participants when implemented with 40% or 20% fidelity when implemented with combined errors.

Differential reinforcement of other behavior (DRO) is a schedule where a learner receives a reinforcer contingent on the absence of a target response, resulting in the reduction of the response (Reynolds, 1961). DRO is used to treat challenging behavior but may be difficult to implement with high procedural fidelity, which refers to the degree to which treatment procedures are executed as designed (Gresham et al., 1993; St. Peter, 2023). For example, in resetting whole‐interval DROs, the implementer is required to constantly monitor for the occurrence of the target response, reset a timer contingent on the response, and provide reinforcement at the end of intervals in which no target response occurred. For implementers who need to divide attention during DRO intervention (e.g., parents in the home, behavior technicians tracking additional responses or presenting demand trials), the resource‐intensive nature of whole‐interval resetting DROs may make it difficult to implement with high procedural fidelity.

There are several components in DRO procedures and, therefore, many different types of errors that implementers may make. Error types can generally be categorized as either *commission errors* or *omission errors*. Commission errors involve the implementation of procedures that are not prescribed by the protocol. Omission errors involve the failure to perform a procedure that is prescribed by the protocol. Additionally, implementers may make both commission and omission errors within a single session (i.e., combined errors).

Both commission and omission errors are possible in DRO. As with many other behavior‐reduction procedures, DRO procedures often specify that the implementer place the target response on extinction (e.g., Vogl & Rapp, 2011; Waters et al., 2009; Wilder et al., 2006). In DROs with extinction for the target response, erroneously reinforcing target behavior is a type of commission error. An additional challenge with DRO is that there are no naturalistic prompts for the implementer to provide a reinforcer. In DRO, the absence of a particular response after the specified time interval is the impetus for reinforcer delivery. The lack of naturalistic prompts to provide a reinforcer, and therefore the reliance on a supplemental clock stimulus such as a timer, may increase the likelihood that earned reinforcers are omitted following a DRO interval in which there was no occurrence of the target response. Omitting reinforcement after a successful DRO interval is a type of omission error that is possible in DRO. These are some examples; other errors during DRO are also possible. For example, other errors of commission could involve providing a reinforcer at the end of an interval in which a target response did occur and errors of omission could involve failing to reset the timer following the occurrence of the target response.

When errors are made when implementing behavioral interventions, treatment effects can be degraded (e.g., Jones & St. Peter, 2022; Jones et al., 2023; St. Peter et al., 2016; St. Peter Pipkin et al., 2010). Generally, relatively lower levels of procedural fidelity result in greater detriments to treatment efficiency and efficacy. This inverse relation has been demonstrated via parametric evaluations of various procedural fidelity levels across a variety of behavioral interventions, including those that address skill acquisition and behavior reduction (e.g., DiGennaro Reed et al., 2011; Leon et al., 2014; Noell et al., 2002; Pence & St. Peter, 2015; St. Peter et al., 2016; Wilder et al., 2006).

In one notable example, St. Peter Pipkin et al. (2010) used a reversal design to evaluate the effects of five procedural fidelity levels (i.e., 100, 80, 60, 40, and 20%) on target and alternative responses during DRA in a human‐operant arrangement. Undergraduate psychology students engaged with a computer program that had two buttons. Clicks to one button served as the target response, and clicks to the second button served as the alternative. The researchers evaluated the effects of procedural fidelity level across different types of errors, including commission, omission, and combined errors. When only omission errors were implemented, the researchers found that rates of the target behavior were low across all procedural fidelity levels. When commission or combined errors were implemented, the researchers found that alternative responses occurred at low rates and target responses occurred at high rates during 40% and 20% procedural fidelity phases; treatment effects were obtained when procedural fidelity ranged from 60% to 100%. Although St. Peter Pipkin et al. found that DRA effects were quite robust with at least 60% fidelity when implemented with commission and combined errors, evidence suggests that the degree to which treatment efficacy is affected by fidelity level may vary across interventions (Brand et al., 2019; Jones & St. Peter, 2022). To date, there is little research on the effects of various procedural fidelity levels during DRO.

A study by Foreman et al. (2023) used a multielement design embedded in a reversal design to assess the effects of different procedural fidelity levels during DRO with commission errors in which the target response was occasionally reinforced. The researchers prepared a human‐operant arrangement during which undergraduates engaged with a computer game with a single button that served as the target response. In baseline, a point was delivered on a fixed‐interval (FI) 3‐s schedule for the target response. In treatment, they implemented a 3‐s DRO schedule and alternated between perfect fidelity (i.e., 100%) and reduced‐fidelity (i.e., 80, 60, 40, and 20%) components each minute. The researchers found that commission errors had no effect for half of the participants (even at 20% fidelity) and had a deleterious effect on the other half of the participants at or below 60% fidelity.

More recently, Hronek and Kestner (2025) conducted a series of three experiments in which they evaluated commission and omission errors during DRO across experiments with different participants. Commission errors were defined as in Foreman et al. (2023), and omission errors involved erroneously omitting a reinforcer that had been earned during the DRO interval. Like Foreman et al., they reinforced the target response on a FI 3‐s schedule in baseline and implemented a 3‐s whole‐interval DRO interval.

In Experiment 1, Hronek and Kestner (2025) conducted a parametric evaluation of commission errors in isolation using a reversal design with six participants. In Experiment 2, the researchers conducted a parametric evaluation of omission errors in isolation with six different participants. In both experiments, participants experienced each DRO condition twice; the researchers presented the reduced‐fidelity conditions in either ascending (i.e., 60, 80, and 100%) or descending (i.e., 100, 80, and 60%) order first and then reversed the order of presentation in the second half of the experiment. In Experiment 3, the researchers presented six different participants with commission errors in isolation and evaluated the effects of reduced‐fidelity DRO under conditions of gradual reduction of procedural fidelity level first (i.e., 100, 80, and 60%) or discontinuous change in procedural fidelity level first (i.e., 100, 60, and 80%) before they presented the other arrangement in the second half of the experiment.

Hronek and Kestner (2025) concluded that both commission and omission errors in isolation were detrimental to treatment efficacy when implemented at 60% fidelity for some participants. They also noted that commission errors were more detrimental than omission errors. Additionally, they found that the sequence of conditions affected the efficacy of DRO when implemented with reduced fidelity. Specifically, experiencing the descending order first had a protective effect on later detriments in procedural fidelity relative to experiencing the ascending order first (i.e., 60, 80, and 100%) and experiencing gradual changes in procedural fidelity (i.e., 100, 80, and 60%) had a protective effect on later detriments relative to discontinuous changes in procedural fidelity (i.e., 100, 60, and 80%).

Foreman et al. (2023) and Hronek and Kestner (2025) provide valuable information regarding the effects of commission and omission procedural fidelity errors on DRO when implemented in isolation. However, it is reasonable to assume that some implementers are likely to engage in a combination of omission and commission errors during DRO. To our knowledge, no one has conducted a parametric analysis of combined errors during DRO. Therefore, the purpose of this study was to parametrically evaluate the effects of procedural fidelity level (i.e., 100, 80, 60, 40, and 20%) using combined errors during DRO.

## METHOD

### Participants

After obtaining approval for this research from the university Institutional Review Board, we enrolled 13 participants in the study after receiving informed consent. Participants were recruited from a local undergraduate psychology program and the general population. Participants were recruited via the university's online participant recruitment platform (Sona Systems Ltd.), email, flyers, and word of mouth. Undergraduate psychology students recruited through Sona received either course credit or a $10 prepaid Visa card for their participation (students self‐selected their preferred form of compensation). All participants recruited outside Sona were offered $10 prepaid Visa cards for their participation. To make it more likely that points served as reinforcers, an additional financial incentive was provided for earning the most points; US$50, $30, and $20 were awarded to the first‐, second‐, and third‐place point earners, respectively (Rey et al., 2020a, 2020b).

To be included in the study, participants had to be at least 19 years old (the age of majority in the state of Nebraska) and had to be able to sit at a computer for up to 120 min (with regular breaks provided throughout the experiment). Current or former students of behavior analysis graduate programs were not eligible to participate in the study. Participants' data would have been excluded under the following conditions: (a) they withdrew consent for their data to be used, (b) they left the experimental area while the computer program was running, (c) their responding did not conform to the contingencies during the reinforcer assessment (Foreman et al., 2023; Rey et al., 2020a, 2020b), or (d) target responding did not decrease by the end of the first 100% DRO condition. Two participants' data were excluded from the analyses based on the final exclusion criterion (P4 and 6; see Supporting Information A).

Therefore, data are presented for 11 participants (10 females, 1 male). Table 1 includes participant sex, age, primary language, race or ethnicity, and recruitment method. Participants ranged in age from 19 to 38 years old (*M* = 24 years). Eight participants were White (73%), two were Hispanic or Latino (18%), and one was both White and Native American (9%). Participants reported speaking English (*n* = 9; 81.81%) and Spanish (*n* = 2; 18.18%) as primary languages. For participants who reported Spanish as their primary language, we did not ask about their English proficiency. However, all these participants were undergraduate students at an English‐speaking institution and conversed with the experimenter in English.

**TABLE 1:** Participant demographics.

Participant	Sex	Age (Years)	Primary language(s)	Race or ethnicity	Recruitment
P1	Female	38	English	White	Undergraduate
P2	Female	19	English	White	Undergraduate
P3	Female	19	English	White	Undergraduate
P5	Male	27	English	White	Undergraduate
P7	Female	23	English	White and Native American	Undergraduate
P8	Female	21	English	White	Undergraduate
P9	Female	20	Spanish	Hispanic or Latino	Undergraduate
P10	Female	27	English	White	Community
P11	Female	20	Spanish	Hispanic or Latino	Undergraduate
P12	Female	19	English	White	Undergraduate
P13	Female	30	English	White	Community

### Setting and apparatus

Research sessions took place in a quiet room with tables and chairs. Each participant was seated at a table with a laptop, external mouse, headphones, and a small collection of fidget items (e.g., pen and paper, Slinky, spinning top, PopIt!). Fidget items were available for participants to manipulate throughout the session to give them something to do during the DRO conditions. During the experiment, participants interacted with a computer program created with PsychoPy, an open‐source software that uses Python syntax and allows researchers to run psychological studies (Peirce et al., 2019). Throughout the experiment, the program displayed a gray screen with a point counter at the top and a single black circle button approximately 1.72 cm in diameter (except during the reinforcer assessment, in which two buttons were present). Only one button was presented to simulate a typical DRO arrangement in which only one target response is explicitly measured and there are no programmed alternative responses, which is consistent with previous evaluations of procedural fidelity in DRO (e.g., Foreman et al., 2023; Hronek & Kestner, 2025). Each time a button was clicked, it momentarily disappeared (about 0.30 s) and reappeared in a different, random location on the screen. Each time a point was awarded, a tone sounded and the point counter briefly turned green. A break screen appeared after the reinforcer assessment and each DRO phase. The break screen displayed the same gray background, but the button disappeared and a brief message indicated that the participant could take a break, if desired, including a 1‐min countdown timer. After 5 s, an orange square button appeared on the screen, which initiated the next phase when clicked. Data were not collected on whether participants took these opportunities for breaks or how much of their allocated break time was used.

### Dependent and independent variables and experimental design

This study evaluated the efficacy of DRO across five levels of procedural fidelity with combined errors. The dependent variable was responses per minute of the target response, defined as clicks on the black button, which served as a proxy for challenging behavior. The independent variable was procedural fidelity level (i.e., 100, 80, 60, 40, and 20%) during DRO with combined errors and was evaluated using a reversal design.

The study consisted of two 25‐min sessions, with an optional 10‐min break between sessions. Each session consisted of five 5‐min components that consisted of an initial baseline phase for 1.5 min followed by an unsignaled change to a DRO phase for the remaining 3.5 min. An optional 1‐min break was provided between each component. Each fidelity level was presented twice for each participant, once per session, to seek within‐participant replication. Due to technical issues (i.e., the computer program contained a bug that prematurely terminated the experiment when a certain number of points was reached), Participant 1 never experienced the second 40% DRO phase, and Participant 3 never experienced the second 40% or 60% DRO phases. Both Participant 1 and Participant 3 experienced only a portion of the second 20% DRO phase. Within each session, the 100% fidelity phase was always presented first, followed by the reduced‐fidelity conditions presented once each in a pseudorandomized order.

### Procedures

#### Informed consent and instructions

Before the session began, participants sat at a desk with a laptop and were asked to set aside watches and cell phones. The experimenter described the upcoming task using the following script, which was read aloud to participants and simultaneously displayed on the laptop screen:

Your goal is to earn as many points as possible. How you earn points may change throughout the experiment. All your points will be visible throughout at the top of the screen. You will be given optional 1‐min breaks every 5 min and a 10‐min break halfway through the experiment (after approximately 30 min). You may skip past the breaks by clicking the button that appears on the screen. You are competing with other people for the most points; the top three point earners in the study will win an [additional]^1^ $1 gift card. Do your best to earn the most points!

We provided an opportunity for participants to review the consent form and ask questions and obtained written informed consent. After participants provided written informed consent, they were asked to complete a brief demographics form. Participants were then seated at the computer for the experiment. Participants could review the instructions displayed on the laptop and clicked anywhere on the screen to begin the experiment.

#### Reinforcer assessment

The purpose of the reinforcer assessment was to determine whether points functioned as reinforcers. For their data to be included, participants had to engage in the target response at greater rates than the alternative response during the last minute of the assessment. All participants met this inclusion criterion. During the reinforcer assessment, the program displayed two circle buttons; clicks on the black button served as the target response, and clicks on the red button served as the alternative response. The target response was reinforced with 1 point on a fixed‐ratio (FR) 1 schedule, and the alternative response had no programmed consequences (i.e., extinction). The reinforcer assessment lasted 3 min.

#### Baseline

In baseline, the target response was reinforced with 1 point on a FI 4‐s schedule.

DRO with 100% fidelity.

A 2‐s whole‐interval resetting DRO was implemented in the DRO 100% fidelity phase; 1 point was delivered contingent on the omission of the target response for the duration of the 2‐s interval. Each occurrence of the target response was placed on extinction and reset the 2‐s DRO interval. We selected a DRO interval that was half of the minimum interreinforcement interval (IRI) in baseline. In clinical research and practice, initial DRO intervals are often set shorter than the mean interresponse time (IRT) observed in baseline (e.g., Repp & Slack, 1977; Ringdahl et al., 2001; Rozenblat et al., 2009; Vollmer & Iwata, 1992), which increases the likelihood that the learner will contact reinforcement during the DRO. To approximate this practice while avoiding excessively short intervals and unreasonably high rates of reinforcement, we based the DRO interval on the minimum IRI rather than the mean IRT.

#### DRO with 80% fidelity

The DRO 80% fidelity phase was identical to the DRO 100% fidelity phase, except that the target response was reinforced on a probabilistic random‐ratio (RR) 5 schedule (commission errors). In other words, a point was “erroneously” provided for about one out of every five responses (i.e., 20% or probability of.20; e.g., Foreman et al., 2023; Hronek & Kestner, 2025; St. Peter Pipkin et al., 2010). To determine if a commission error would be made, following each click, the computer program generated a random integer between 1 and 5, each with equal probability. If the integer was 1, the target response was reinforced with a point; if the integer was 2–5, no point was delivered.

In this phase, all responses continued to result in the resetting of the DRO interval, including those that occasioned a commission error (i.e., those that were reinforced with a point). Additionally, the omission of the target response was reinforced on a probabilistic random omission interval in which four out of five successful DRO intervals were reinforced (i.e., 80%; probability of .80; Hronek & Kestner, 2025). In other words, a point was delivered for four out of every five, 2‐s intervals in which the target response was omitted (omission errors).

#### DRO with 60% fidelity

This phase was identical to the DRO 100% fidelity phase, except that the target response was reinforced on a RR 2.50 schedule (i.e., 40%; probability of .40) and the omission of the target response was reinforced on three out of five opportunities on a random schedule (i.e., 60%; probability of .60). To determine if a commission error would be made, following each click, the computer program generated a random integer between 1 and 5, each with equal probability. If the integer was 1 or 2, the target response was reinforced with a point; if the integer was 3–5, no point was delivered.

#### DRO with 40% fidelity

This phase was identical to the DRO 100% fidelity phase, except that the target response was reinforced on a RR 1.67 schedule (i.e., 60%; probability of .60) and the omission of the target response was reinforced on two out of five opportunities (i.e., 40%; probability of .40). To determine if a commission error would be made, following each click, the computer program generated a random integer between 1 and 5, each with equal probability. If the integer was 1–3, the target response was reinforced with a point; if the integer was 4 or 5, no point was delivered.

#### DRO with 20% fidelity

This phase was identical to the DRO 100% fidelity phase, except that the target response was reinforced on a RR 1.25 schedule (i.e., 80%; probability of .80) and the omission of the target response was reinforced on one out of five opportunities (i.e., 20%; probability of .20). To determine if a commission error would be made, following each click, the computer program generated a random integer between 1 and 5, each with equal probability. If the integer was 1–4, the target response was reinforced with a point; if the integer was 5, no point was delivered.

#### Postexperiment

After participants completed the experiment, they were thanked for their time and issued either their class credit via Sona or provided with a $10 prepaid Visa card. After all participants completed the experiment, the experimenters contacted the top three point earners to issue the $50, $30, and $20 bonus gift cards.

### Statistical analysis

We performed statistical analyses to assess differences in the percentage of change in responding from baseline across the five levels of procedural fidelity using SPSS (v 29.0.2). Percentage of change from baseline scores was compared across the 100% fidelity conditions and all four reduced‐fidelity levels for each participant. Percentage of change from baseline for each DRO phase was defined conservatively as the mean number of clicks per 30 s during the entire DRO phase divided by the mean number of clicks per 30 s during the preceding baseline phase, subtracted from 1. This measure is conservative because it included data from periods during which responding was in transition and those during which it had reached terminal response suppression, thereby reducing the effect size relative to an analysis based on terminal performance.

For each participant, we calculated the mean percentage of change from baseline across both presentations of each procedural fidelity level; therefore, each participant contributed one score for each of the five fidelity levels. In the case that participants completed only one exposure to a given phase (i.e., P1 and P3), only data from the completed phase were included. Planned pairwise comparisons were conducted using Wilcoxon signed‐rank tests, a nonparametric alternative to the paired‐samples *t* test, to compare the 100% fidelity DRO conditions with all reduced‐fidelity DRO conditions. A Bonferroni correction was applied to adjust the α level due to multiple comparisons. The adjusted significance threshold was set at α = .0125, based on the formula α(adjusted) = α(desired) / *h*, where α(desired) = .05 and *h* is the total number of pairwise comparisons (*h* = 4). Finally, effect sizes for each pairwise comparison were calculated as |*r*| = *z* / √*n*, where *n* is the number of pairwise comparisons (i.e., 11).

### Obtained fidelity

We programmed the computer to implement DRO with 80, 60, 40, and 20% fidelity, but the obtained procedural fidelity for any given reduced‐fidelity DRO session may have varied from the programmed fidelity level as a function of the RR schedule or participant responding. Therefore, we calculated the obtained fidelity level for each reduced‐fidelity DRO phase. Obtained fidelity for extinction of the target response (i.e., the commission errors component of the combined errors) was calculated as the number of target responses that were not followed by a point divided by the number of target responses that were not followed by a point plus the number of target responses followed by a point (Hronek & Kestner, 2025). Obtained fidelity for providing a point at the end of a successful DRO interval (i.e., omission errors component of the combined errors) was calculated as the number of successful DRO intervals that resulted in a point divided by the number of successful DRO intervals that resulted in a point plus the number of successful DRO intervals that did not result in a point (Hronek & Kestner, 2025).

## RESULTS

### Obtained fidelity levels

Obtained fidelity values across reduced DRO fidelity phases (i.e., 80, 60, 40, and 20%) are presented in Table 2 for the 11 participants included in the analysis and in Supporting Information B for the two excluded participants. Obtained fidelity for commission and omission errors was always 100% in baseline and 100% fidelity phases (and not included in Table 2). Median obtained fidelity for commission and omission errors was 86.04% (range: 57.14%–100%) and 80.39% (range: 0%–84.95%), respectively, in the 80% DRO phase; 61.22% (range: 0%–100%) and 62.05% (range: 48.54%–73.53%), respectively, in the 60% DRO phase; 40% (range: 0%–100%) and 38.84% (range: 0%–52%), respectively, in the 40% DRO phase; and 18.46% (range: 8.57%–100%) and 18.48% (range: 0%–100%), respectively, in the 20% DRO phase.

**TABLE 2:** Obtained fidelity values for reduced‐fidelity conditions.

Participant	Programmed fidelity level							
	80%		60%		40%		20%	
	Sess. 1	Sess. 2	Sess. 1	Sess. 2	Sess. 1	Sess. 2	Sess. 1	Sess. 2
P1	86.36 (82.89)	N/A (78.85)	76.92 (61.86)	71.43 (60.64)	41.02 (N/A)	–	14.61 (N/A)	14.74 (11.11)
P2	100 (80.58)	100 (75)	50 (70.87)	0 (48.54)	100 (46.15)	50 (38.46)	14.81 (15.91)	100 (16.50)
P3	79.51 (0)	81.18 (66.67)	58.80 (N/A)	–	37.99 (N/A)	–	21.36 (N/A)	15.69 (N/A)
P5	77.78 (75.76)	82.14^a^	66.67 (67.33)	100 (55.90)	37.64 (0)	38.97 (N/A)	18.37 (N/A)	18.46 (100)
P7	100 (80.39)	57.14 (81.37)	56.52 (49.45)	63.64 (62.24)	31.58 (38.30)	42.31 (32.26)	18.18 (18.48)	20.53 (0)
P8	75 (79.79)	100 (77.67)	75.86 (64.04)	52.63 (61.70)	41.80 (0)	45.57 (46.67)	25.30 (18.03)	16.16 (N/A)
P9	100 (82.52)	80.28 (75.56)	57.14 (64)	64.81 (55.43)	42.10 (35.05)	38.46 (51.61)	8.57 (19.77)	17.36 (25)
P10	100 (77.67)	100 (78.85)	N/A (66.35)	100 (53.85)	66.67 (42.72)	60 (52)	30 (22.22)	20.83 (18.48)
P11	73.08 (76.04)	57.14 (84.95)	66.67 (64.77)	66.67 (73.53)	32.26 (33.33)	40 (48)	21.43 (30.77)	21.88 (18.06)
P12	85.71 (84)	N/A (79.81)	50 (63.37)	0 (55.77)	33.33 (38.84)	N/A (45.19)	23.26 (16.05)	N/A (24.04)
P13	N/A (82.69)	100 (79.81)	50 (62.50)	0 (61.54)	40 (39.81)	0 (37.26)	16.67 (20.20)	33.33 (17.71)
Range	73.08–100 (0–84)	57.14–100 (66.67–84.95)	50–76.92 (49.45–70.87)	0–100 (48.54–73.53)	31.58–100 (0–46.15)	0–60 (32.26–52)	8.57–30 (15.91–30.77)	14.74–100 (0–100)
Mean	87.74 (72.94)	84.21 (77.85)	60.86 (63.45)	51.92 (58.91)	45.85 (30.47)	39.41 (43.93)	19.32 (20.18)	27.90 (25.66)
Median	86.04 (80.39)	82.14 (78.85)	57.97 (64.02)	64.23 (58.27)	40 (38.30)	41.16 (45.93)	18.37 (19.12)	19.50 (18.06)
Overall median	85.71 (79.79)		61.22 (62.05)		40 (38.84)		18.46(18.48)	

*Note*: Sess. = session. Numerals outside parentheses indicate the fidelity level for placing target response on extinction (i.e., commission errors). Numerals inside parentheses indicate the fidelity level for providing a point contingent on a successful DRO interval (i.e., omission errors). N/A indicates no opportunity to implement an error. Dashes (−) indicate the phases that P1 and P3 did not experience.

^a^ Due to technical errors with data outputs, there are no obtained fidelity data of the latter type for the second 80% DRO phase for P5.

It is important to note that, in many instances where obtained fidelity level varied meaningfully from programmed fidelity level, the variation in obtained fidelity was a function of minimal opportunities to make procedural fidelity errors due to participant responding. For example, Participant 2 engaged in only one target response during the first 40% fidelity phase. Because the RR schedule resulted in omitting reinforcement for this singular response, the obtained fidelity level was 100% for commission errors despite the programmed 40% fidelity level. Also note that there were some phases without any opportunities to make either commission errors or omission errors, denoted by “N/A” in Table 2. Specifically, some participants engaged in zero target responses during the DRO phase, resulting in no opportunity for commission errors (e.g., P13 during the 80% DRO phase in the first session). Additionally, some DRO phases passed without a 2‐s interval in which the target response was absent, resulting in no opportunity for omission errors (e.g., P5 during the 40% DRO phase in the second session).

### Within‐subject data

**FIGURE 1:**
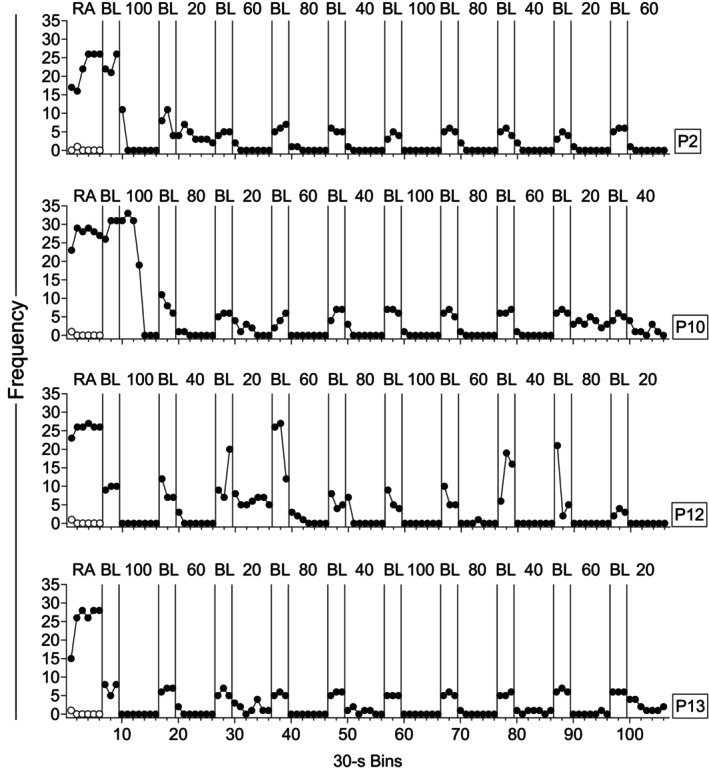
Participants with majority reduction. RA = reinforcer assessment; BL = baseline. Closed data points represent clicks on the target button. Open data points represent clicks on the alternative button.

**FIGURE 2:**
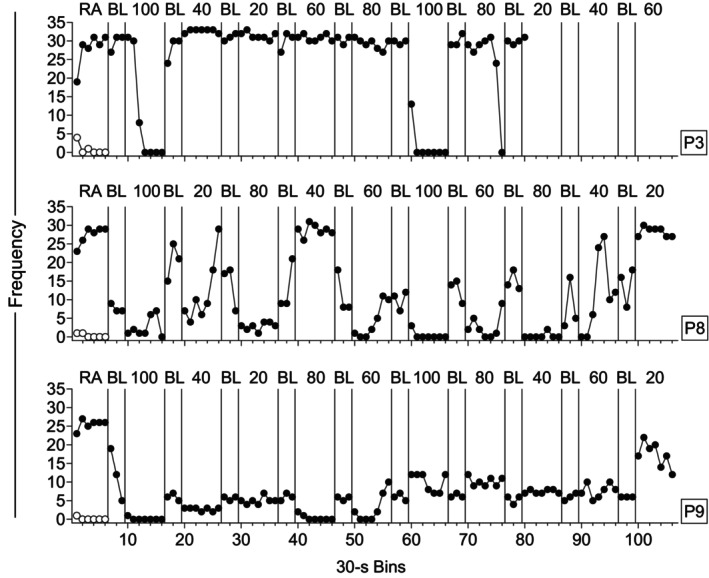
Participants with reduction in the 100% and 80% DRO phases. RA = reinforcer assessment; BL = baseline. Closed data points represent clicks on the target button. Open data points represent clicks on the alternative button. Due to technical errors with the computer program, P3 did not experience part of the second 20% DRO phase or any of the subsequent phases.

**FIGURE 3:**
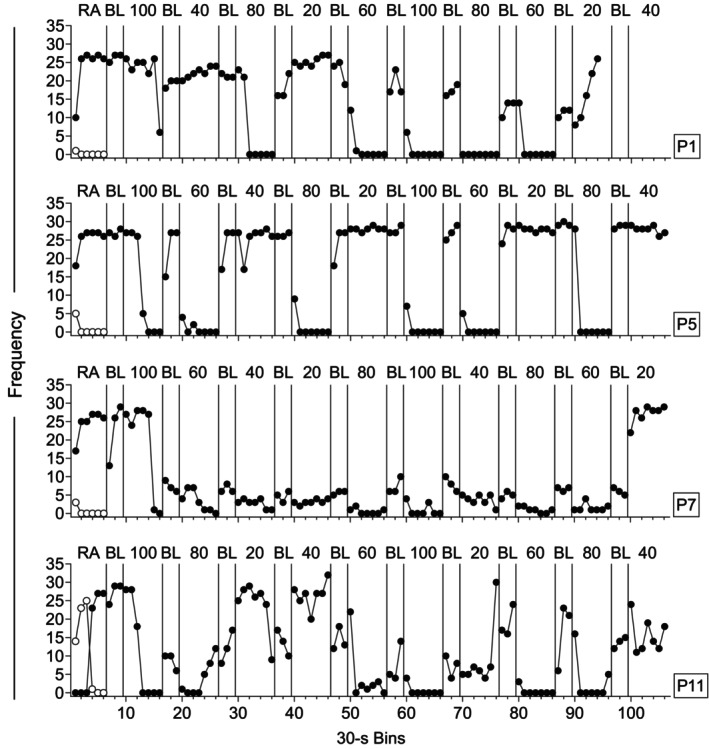
Participants with reduction in the 100, 80, and 60% DRO phases. RA = reinforcer assessment; BL = baseline. Closed data points represent clicks on the target button. Open data points represent clicks on the alternative button. Due to technical errors with the computer program, P1 did not experience part of the second 20% DRO phase or any of the subsequent phases.

Figures 1, 2, 3 display the rate of responding across each phase of the study for each participant; data sets are incomplete for two participants (P1 and P3) who did not experience all phases due to technical errors with the computer program. Supporting Information C displays the reversal graphs for the two excluded participants. Within‐subject replication was assessed based on visual inspection of the reversal graphs, which included consideration of level, trend, and variability of responding.

The DRO reduced target responding during at least one 100% DRO phase for all 11 participants (100%); note that reduced responding during the first 100% condition was an inclusion criterion (see Supporting Information C for participants who did not meet this criterion). This effect was replicated across both 100% DRO phases for 10 participants (P1, P2, P3, P5, P7, P8, P10, P11, P12, and P13; 90.91%). The DRO reduced target responding in at least one 80% DRO phase for all 11 participants (100%) and replicated across both 80% DRO phases for eight of those participants (P1, P2, P5, P7, P10, P11, P12, and P13; 72.73%). The DRO reduced target responding in at least one 60% DRO phase for eight of 10 participants (P1, P2, P5, P7, P10, P11, P12, and P13; 80%^2^) and replicated across both phases for all eight of those participants (100%). The DRO reduced target responding in at least one 40% DRO phase for four participants (P2, P10, P12, and P13; 44.44%^3^) and across both 40% DRO phases for all four of those participants (100%). The DRO reduced target responding in at least one 20% DRO phase for four participants (P2, P10, P12, and P13; 36.36%^4^) and replicated across both phases for one of those participants (P13; 25%). Absent effects were replicated across both 60% DRO phases for two participants (P8, P9; 20%^5^), across both 40% DRO phases for five participants (P5, P7, P8, P9, P11; 55.56%^6^), and across both 20% DRO phases for seven participants (P1, P3, P5, P7, P8, P9, P11; 63.64%^7^).

#### Reduction in the 100, 80, 60, 40, and 20% DRO phases

Figure 1 displays data for the four participants (P2, P10, P12, and P13) who showed a reduction in the target response across almost all DRO phases. For P2, P10, and P12, the target response was eliminated at the end of each 3.5‐min DRO condition in all phases except for one 20% DRO phase. For P13, the target response was at zero or near‐zero levels at the end of each DRO condition in all phases other than both 20% DRO phases, although the frequency of responding was reduced relative to baseline.

#### Differential reduction

Figures 2 and 3 display data for the remaining seven participants (P1, P3, P5, P7, P8, P9, and P11), who showed a reduction in target responses across some but not all DRO phases. Among these participants, the DRO was generally more efficacious at higher fidelity levels (i.e., 100% and 80%), somewhat less efficacious at moderate fidelity levels (i.e., 60%), and generally inefficacious at low fidelity levels (i.e., 40% and 20%).

##### Reduction in the 100% and 80% DRO phases

Figure 2 shows data for P3, P8, and P9, for whom target responding was reduced in the 100% and 80% DRO phases. For P3, target responding was eliminated by the end of both 100% DRO phases and the second 80% DRO phase. For P8, target responding was eliminated by the end of both 100% DRO phases and the second 80% DRO phase and was reduced relative to baseline in the first 80% DRO condition. For P9, responding was eliminated at the end of the first 100% DRO phase and the first 80% DRO phase only. For all three participants, responding was at or above baseline levels by the end of the 60, 40, and 20% DRO phases.

##### Reduction in the 100, 80, and 60% DRO phases

Figure 3 shows data for P1, P5, P7, and P11, for whom target responding was reduced or eliminated in the 100, 80, and 60% DRO phases. For P1, responding persisted at baseline rates for the first 3 min of the first DRO phase and decreased sharply in the last 30 s. Responding was eliminated in the second 100% DRO phase and both 80% and 60% DRO phases and remained at or above baseline levels in the 40% and 20% DRO phases. For P5, responding was eliminated at the end of both occurrences of the 100, 80, and 60% DRO phases and remained at baseline levels in both 40% and 20% DRO phases. For P7, responding was reduced but not necessarily eliminated in the 100, 80, and 60% DRO phases. They also showed slightly decreased rates of responding in both 40% DRO phases and rates of responding at or well above baseline levels in both 20% DRO phases. P11 also showed reduced responding in the 100, 80, and 60% DRO phases. The frequency of responding increased at the end of the 80% DRO phase in both sessions following over a minute of response elimination. Responding occurred around or above baseline levels in both 40% and 20% DRO phases.

### Aggregate results

**FIGURE 4:**
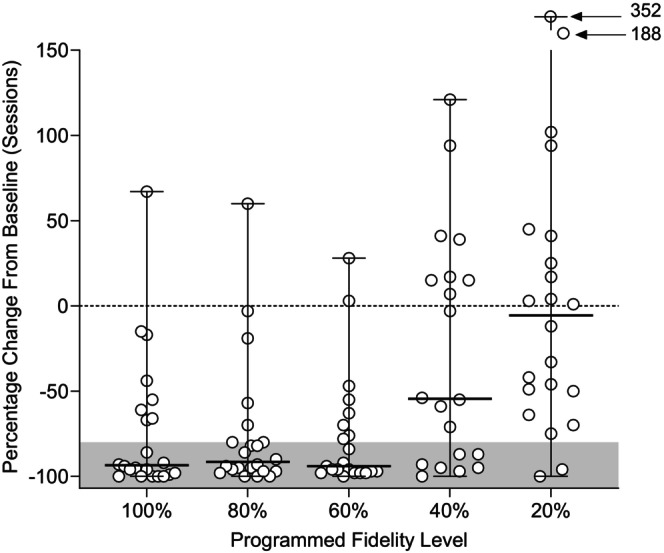
Aggregate percentage of change from baseline. The horizontal dashed line indicates mean rate of responding equivalent to that in the previous baseline phase. Bold horizontal lines within each fidelity level indicate the median of all sessions. Whiskers indicate the minimum and maximum values across all sessions within each fidelity level. The gray shaded area represents at least 80% reduction from baseline.

Figure 4 displays the percentage of change in the rate of responding from baseline to each DRO phase aggregated across all participants. We defined DRO phases as efficacious if mean rate of responding across the entire DRO phase was reduced at least 80% from the previous baseline phase (indicated by the gray shaded region in the figure). Across participants, the DRO was efficacious in 14 out of 22 sessions (63.64%) in the 100% fidelity phase (which was presented first in each session), 17 of 22 sessions (77.27%) in the 80% phase, 13 of 21 sessions (61.90%)^8^ in the 60% phase, 7 out of 20 sessions (35%)^9^ in the 40% phase, and 2 out of 22 sessions (9.09%)^10^ in the 20% phase.

Bolded horizontal lines in Figure 4 indicate the median percentage of change from baseline across all sessions for each fidelity level. Overall, the 100, 80, and 60% phases were the most efficacious (median = −93.50, −91.50, and − 94% change, respectively), the 40% phase was only somewhat efficacious (i.e., resulted in only a partial reduction from baseline; median = −54.50% change), and the 20% phase was inefficacious (i.e., resulted in baseline levels of responding; median = −5.50% change). However, there is notable variation in the percentage of change from baseline scores. All procedural fidelity levels nearly eliminated responding in at least one case across participants, and all produced rates of responding approximately equal to or greater than baseline rates in at least one case. The degree of variability across fidelity levels indicates that changes in responding were not uniformly associated with programmed fidelity levels across participants. Although median values summarize overall trends, individual data reveal within‐ and between‐participant variability, particularly at intermediate fidelity levels (e.g., 60% and 40%).

#### Statistical analysis

Pairwise comparisons using the Wilcoxon signed‐rank test with Bonferroni correction applied indicate a statistically significant difference and large effect size between 20% DRO versus 100% DRO (see Table 3). The comparison of 40% DRO versus 100% DRO also showed a large effect size but only approached statistical significance; this suggests a potentially meaningful difference that may not have reached significance due to limited sample size or the conservative Bonferroni correction. Statistically significant differences were not detected for 60% DRO versus 100% DRO and 80% DRO versus 100% DRO, and the effect sizes were small. Overall, the results of statistical analyses support the conclusions reached via visual inspection, which suggest DRO implemented with 100, 80, or 60% fidelity produced comparable results. However, the efficacy of the DRO was somewhat degraded at 40% fidelity and was generally inefficacious at 20% fidelity.

**TABLE 3:** Results of the Wilcoxon signed‐rank tests.

Comparison	*Z*	*p*	*|r|*
80% vs. 100%	−.622	.534	.188
60% vs. 100%	−.533	.594	.161
40% vs. 100%	−2.223	.026	.670^†^
20% vs. 100%	−2.934	.003*	.885^†^

* *p* < .0125.

^†^ large effect size.

## DISCUSSION

DRO interventions are used to address challenging behavior but can be difficult to implement because DRO requires continuous observation and delivery of a reinforcer based on events that may not be salient (i.e., the absence of the target response over a defined period). Many types of procedural fidelity errors are possible in DRO, and it is possible that implementers will make multiple types of errors. To better understand the degree to which errors in the implementation of DRO diminish treatment efficacy, we conducted a parametric evaluation of procedural fidelity level with combined commission and omission errors during DRO in a human‐operant arrangement. Combined errors consisted of periodically reinforcing target responses (i.e., commission errors) and withholding reinforcement contingent on successful DRO intervals in which the participant did not engage in the target response (i.e., omission errors). Results analyzed via visual inspection and statistical analyses showed that DRO was efficacious for the majority of participants when implemented with 100, 80, and 60% fidelity and for only a few participants when implemented with 40% or 20% fidelity with combined commission and omission errors. However, these findings should be considered tentatively given issues with within‐subject replication and concerns with experimental control in the reversal designs.

To our knowledge, only two other peer‐reviewed studies have evaluated DRO with fidelity errors, and neither evaluated combined commission and omission errors. Foreman et al. (2023) evaluated DRO implemented with commission errors under various levels of fidelity (i.e., 100, 80, 60, 40, and 20%) and found that DRO was efficacious at all fidelity levels for half of the six participants in their study. For the other half, the DRO was efficacious at or above 80%. Hronek and Kestner (2025) assessed the effects of both commission and omission errors in isolation across three fidelity levels (i.e., 100, 80, and 60% fidelity) across studies with different participants. Across Experiments 1 and 3, these authors found that commission errors were detrimental to treatment efficacy for eight of 12 participants when implemented in isolation with 80% or 60% fidelity. These detrimental effects were especially prevalent among participants who experienced reduced‐fidelity conditions prior to full‐fidelity conditions or who experienced fidelity changes occurring in a discontinuous fashion (i.e., 100, 60, and 80%) rather than a gradual fashion (i.e., 100, 80, and 60%). In Experiment 2, two of six participants showed increased rates of responding in the reduced‐fidelity phases when implemented at 80% or 60% fidelity with omission errors. Unlike with commission errors, the sequence in which full‐ and reduced‐fidelity conditions were introduced did not appear to influence efficacy of the DRO.

In the present study, the DRO was more often efficacious at lower levels of fidelity than in Foreman et al. (2023) and Hronek and Kestner (2025; i.e., treatment effects were replicated in both 60% DRO phases for eight participants). This discrepancy may be due to the different DRO intervals used in each study. Foreman et al. (2023) and Hronek and Kestner (2025) employed FI 3‐s baselines and 3‐s DROs, whereas we implemented a FI 4‐s baseline and 2‐s DRO. We selected a DRO interval that was half of the minimum IRI in baseline to approximate how DRO intervals are often selected relative to baseline IRT in applied contexts. Existing research suggests that initial DRO intervals that are shorter than the mean IRT in baseline are more efficacious than DRO intervals that are longer than the mean IRT in baseline (Repp & Slack, 1977; Rozenblat et al., 2009). In these arrangements, participants are more likely to contact the DRO contingency earlier in treatment, resulting in more efficient behavior reduction. Compared with those of Foreman et al. and Hronek and Kestner, the DRO intervals in the present study likely allowed participants to more readily contact the DRO contingency, resulting in the more efficacious behavior reduction during reduced‐fidelity phases.

Within the present study, different patterns of responding were observed across participants; four (36.36%) showed reductions in target responding across all the DRO phases, and seven (63.64%) showed differentiated responding across the DRO phases. Based on visual inspection, within‐subject replication was not obtained for one to two fidelity levels in two thirds of participants (*n* = 6; 66.67%). However, across all participants and fidelity levels, within‐subject replication was obtained in 45 of 52 comparisons (86.54%).

The short phase durations sometimes resulted in phase changes that occurred while responding was in a transition state (i.e., not stable). Short phase durations were used to collect data efficiently within a single session, and this limits our ability to observe steady‐state responding in the reversal across multiple fidelity levels. It is unclear whether the same patterns of responding would emerge had conditions been maintained until steady‐state responding was achieved. The present data may capture participants' initial sensitivity to changes in contingencies rather than long‐term changes that may be expected during extended exposure to DRO implemented at various levels of procedural fidelity. Using longer phases or otherwise ensuring stability in responding prior to making phase changes may have enhanced experimental control and better supported within‐subject replication.

Furthermore, the short phase durations may have contributed to variability as a result of the obtained fidelity. Because both commission and omission errors were contingent on participant responding, the obtained fidelity varied at times from the programmed fidelity. Longer phase durations may have resulted in fewer phases where either commission or omission errors were not experienced. The use of alternative methods for determining when errors are presented might also have promoted obtained fidelity levels that more closely approximated programmed fidelity levels, such as using nonprobabilistic RR schedules instead of probabilistic RR schedules or programming the computer to maintain the programmed fidelity level across smaller segments of the DRO phase.

Given the translational arrangement of this investigation, these results should be interpreted cautiously. As with all human‐operant studies, external validity is limited due to the analog arrangement; it is possible that findings may differ in applied contexts with clinical populations, target behaviors that require higher response effort, longer IRTs, longer DRO intervals, more potent reinforcers, and longer or more complex histories of reinforcement. Further research may seek to directly evaluate the influence of these and other variables that might influence the efficacy of DRO under conditions of reduced fidelity, such as rate of responding and rate of reinforcement in baseline.

In addition to potential issues of generalization, some methodological limitations are also worth noting. Although we presented the reduced‐fidelity phases in a pseudorandomized order within and across participants, presenting the 100% fidelity phases first might have influenced our findings relative to those of Foreman et al. (2023) or Hronek and Kestner (2025), who did not always present the 100% DRO first. It is possible that results would have differed had the 100% fidelity phases been presented elsewhere in the sequence, as suggested by data obtained by Hronek and Kestner.

Future researchers should evaluate other types of errors not yet evaluated in the literature, including errors where a point is erroneously delivered at the end of a DRO interval in which a target response occurred. To our knowledge, no studies have described common procedural fidelity errors during DRO in practice, and data to this effect may better inform future procedural fidelity research in DRO (see Carroll et al., 2013, for an evaluation of errors during discrete‐trial instruction in applied contexts). Additionally, studies could investigate how procedural fidelity errors affect the maintenance of behavior reduction obtained under high‐fidelity conditions. Further evaluations of procedural fidelity errors should be conducted across different variations of DRO that are unexamined in this body of literature, including DROs without extinction for the target response, non‐resetting DROs, and momentary DROs.

In conclusion, the findings of this study add to the literature on procedural fidelity and DRO in several ways. To our knowledge, this is the first study to conduct a parametric evaluation of DRO using combined commission and omission errors. This is also the first parametric evaluation of procedural fidelity in DRO to use DRO intervals that were shorter than the minimum baseline IRI, which more closely approximates how initial DRO intervals are selected in clinical contexts. The short‐duration phases of the within‐session parametric evaluation allowed for efficient evaluation of many levels of procedural fidelity with combined errors but produced limitations related to steady‐state responding and experimental control, which limits the degree to which the present findings represent those that may be expected under longer duration exposure to various procedural fidelity levels in DRO. Future researchers should consider designs that permit extended exposure to reduced‐fidelity DRO and limit the risk of sequence effects to better understand how procedural fidelity level affects the efficacy of DRO for behavior reduction.

## AUTHOR CONTRIBUTIONS

The first author contributed to conceptualization, formal analysis, funding acquisition, investigation, methodology, software, visualization, and original draft preparation. The second author contributed to conceptualization, formal analysis, funding acquisition, methodology, supervision, and manuscript review and revisions. The third author contributed to methodology and software. The fourth author contributed to formal analysis, and manuscript review, and revisions.

## CONFLICT OF INTEREST STATEMENT

Andrew R. Craig was on the SEAB committee that provided funding for this research but was not a collaborator at the time of funding decisions.

## ETHICS APPROVAL

This study received Institutional Review Board approval and was conducted in accordance with established ethical guidelines for the treatment of human participants.

## Supporting information


**Supporting Information A** *Demographics of Excluded Participants*
**Supporting Information B**. *Obtained Fidelity Values for Reduced‐Fidelity Conditions for Excluded Participants*
**Supporting Information C**. *Reversal Data for Excluded Participants*

## Data Availability

The data that support the findings of this study are available from the corresponding author upon reasonable request. Supporting Information A–C include demographic data, obtained fidelity values, and reversal graphs for excluded participants.

## Footnotes

^1^
The word “additional” was omitted if participants received course credit instead of the $10 gift card.

^2^
P3 did not experience the 60% phase in the second session.

^3^
P1 and P3 did not experience the 40% phase in the second session.

^4^
Note that P1 and P3 experienced only a portion of the 20% phase in the second session.

^5^
P3 did not experience the 60% phase in the second session.

^6^
P1 and P3 did not experience the 40% phase in the second session.

^7^
Note that P1 and P3 experienced only a portion of the 20% phase in the second session.

^8^
P3 did not experience the 60% phase in the second session.

^9^
P1 and P3 did not experience the 40% phase in the second session.

^10^
Note that P1 and P3 experienced only a portion of the 20% phase in the second session.
