# Supplementary material for: Parametric evaluation of procedural fidelity level with combined errors during differential reinforcement of other behavior
Source: J Appl Behav Anal. 2026 Aug 17;59(4):e70077. doi: 10.1002/jaba.70077 (PMC13481847; doi:10.1002/jaba.70077)
Supplement: Supplementary file 1 [file JABA-59-0-s001.docx]

**Supporting Information**

**Supporting Information A**

*Demographics of Excluded Participants*

| Participant | Sex | Age (Years) | Primary Language(s) | Race or Ethnicity | Recruitment |
| --- | --- | --- | --- | --- | --- |
| P4 | Female | 19 | English | Black or African American | Undergraduate |
| P6 | Female | 21 | English and Vietnamese | Asian | Undergraduate |

**Supporting Information B**

*Obtained Fidelity Values for Reduced-Fidelity Conditions for Excluded Participants*

| Participant | Programmed Fidelity Level | | | | | | | |
| --- | --- | --- | --- | --- | --- | --- | --- | --- |
|  | 80% | | 60% | | 40% | | 20% | |
|  | Sess. 1 | Sess. 2 | Sess. 1 | Sess. 2 | Sess. 1 | Sess. 2 | Sess. 1 | Sess. 2 |
| P4 | 86.42 (81.40) | 100 (81.73) | 58.89 (57.53) | 64.70 (61.11) | 37.65 (34.38) | 43.10 (38.16) | 22.89 (21.21) | 24.05 (29.70) |
| P6 | 80.81 (100) | 76.43 (90.91) | 55.56 (N/A) | 61.82 (37.50) | 46.04 (N/A) | 39.25 (33.33) | 18.90 (0) | 18.86 (25) |

*Note*. Sess. = session. Numerals outside parentheses indicate the fidelity level for placing target response on extinction (i.e., commission errors). Numerals inside parentheses indicate the fidelity level for providing a point contingent on a successful differential reinforcement of other behavior (DRO) interval (i.e., omission errors). N/A indicates no opportunity to implement an error.

**Supporting Information C**

*Reversal Data for Excluded Participants*

*Note*. RA = reinforcer assessment; BL = baseline. Closed data points represent clicks on the target button. Open data points represent clicks on the alternative button.
